# (*Z*)-Methyl 2-bromo­methyl-3-(2-chloro­phen­yl)acrylate

**DOI:** 10.1107/S1600536811039663

**Published:** 2011-09-30

**Authors:** R. Madhanraj, S. Vijayakumar, R. Selvakumar, M. Bakthadoss, S. Murugavel

**Affiliations:** a Department of Physics, Ranipettai Engineering College, Thenkadapathangal, Walaja 632 513, India; b Department of Physics, Sri Balaji Chokkalingam Engineering College, Arni, Thiruvannamalai 632 317, India; c Department of Organic Chemistry, University of Madras, Maraimalai Campus, Chennai 600 025, India; d Department of Physics, Thanthai Periyar Government Institute of Technology, Vellore 632 002, India

## Abstract

In the title compound, C_11_H_10_BrClO_2_, the dihedral angle between the benzene ring and the plane of the acrylate unit is 62.1 (1)°. The crystal packing is stabilzed by inter­molecular C—H⋯O hydrogen bonds and C—Cl⋯π inter­actions [Cl⋯centroid = 3.829 (1) Å and C—Cl⋯centroid = 165.3 (1)°].

## Related literature

For background to the applications of acrylates, see: de Fraine & Martin (1991 ▶); Zhang & Ji (1992 ▶). For related structures, see: Wang *et al.* (2011 ▶); Ren *et al.* (2008 ▶). For hydrogen-bond motifs, see: Bernstein *et al.* (1995 ▶).
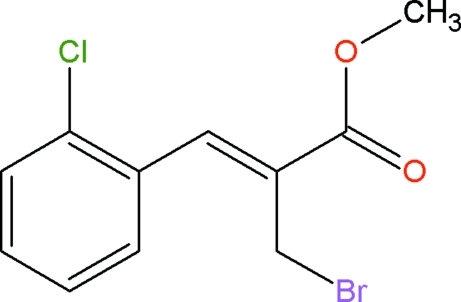


## Experimental

### 

#### Crystal data

C_11_H_10_BrClO_2_
*M* *_r_* = 289.55
Monoclinic, 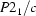

*a* = 10.0657 (7) Å
*b* = 10.2174 (7) Å
*c* = 11.3598 (7) Å
β = 97.649 (2)°
*V* = 1157.91 (13) Å^3^
*Z* = 4
Mo *K*α radiation
μ = 3.76 mm^−1^
*T* = 293 K
0.24 × 0.22 × 0.16 mm

#### Data collection

Bruker APEXII CCD diffractometer
Absorption correction: multi-scan (*SADABS*; Sheldrick, 1996 ▶) *T*_min_ = 0.390, *T*_max_ = 0.548
14580 measured reflections
3336 independent reflections
2139 reflections with *I* > 2σ(*I*)
*R*_int_ = 0.034

#### Refinement

*R*[*F* ^2^ > 2σ(*F* ^2^)] = 0.034
*wR*(*F* ^2^) = 0.089
*S* = 0.99
3336 reflections
137 parameters
H-atom parameters constrained
Δρ_max_ = 0.57 e Å^−3^
Δρ_min_ = −0.54 e Å^−3^

### 

Data collection: *APEX2* (Bruker, 2004 ▶); cell refinement: *APEX2* and *SAINT* (Bruker, 2004 ▶); data reduction: *SAINT* and *XPREP* (Bruker, 2004 ▶); program(s) used to solve structure: *SHELXS97* (Sheldrick, 2008 ▶); program(s) used to refine structure: *SHELXL97* (Sheldrick, 2008 ▶); molecular graphics: *ORTEP-3* (Farrugia, 1997 ▶); software used to prepare material for publication: *SHELXL97* and *PLATON* (Spek, 2009 ▶).

## Supplementary Material

Crystal structure: contains datablock(s) global, I. DOI: 10.1107/S1600536811039663/kj2189sup1.cif

Structure factors: contains datablock(s) I. DOI: 10.1107/S1600536811039663/kj2189Isup2.hkl

Supplementary material file. DOI: 10.1107/S1600536811039663/kj2189Isup3.cml

Additional supplementary materials:  crystallographic information; 3D view; checkCIF report

## Figures and Tables

**Table 1:** Hydrogen-bond geometry (Å, °)

*D*—H⋯*A*	*D*—H	H⋯*A*	*D*⋯*A*	*D*—H⋯*A*
C2—H2⋯O1^i^	0.93	2.48	3.373 (3)	162

Symmetry code: (i) 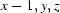.

**Fig. 1.**
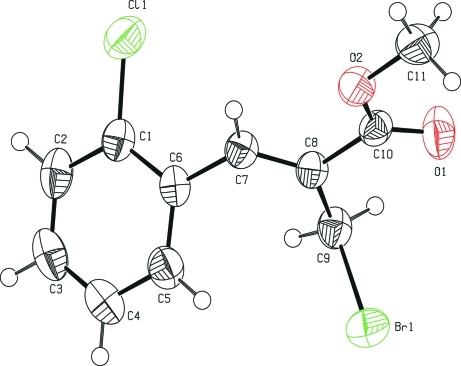
The molecular structure of the title compound with the atom numbering scheme. Displacement ellipsoids are drawn at the 50% probability level.

**Fig. 2.**
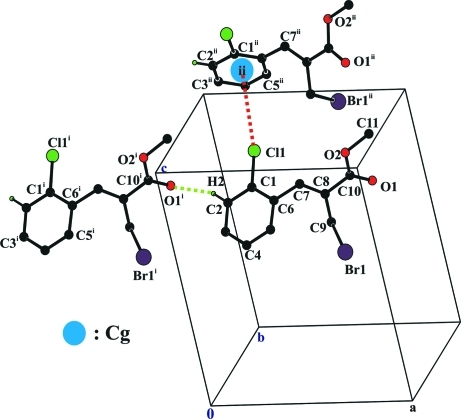
A view of the intermolecular C—H···O and C—Cl···π interactions (dotted lines) in the crystal structure of the title compound. H atoms not involved in intermolecular interactions were omitted. Cg denotes centroid of the C1—C6 benzene ring [Symmetry code: (i) -1+x, y, z; (ii) x, 1/2-y, 1/2+z.]

## supplementary crystallographic information

### Comment

Acrylate and its derivatives are important compounds because of their agrochemical and medical applications (de Fraine *et al.,* 1991; Zhang & Ji, 1992). We report herein the crystal structure of the title compound (Fig. 1). The acrylate plane (C7/C8/C10/C11/O1/O2) forms a dihedral angle of 62.1 (1)° with the benzene ring (C1—C6). The geometric parameters of the title molecule agree well with those reported for similar structures (Wang *et al.,* 2011, Ren *et al.,* 2008).

The molecule is stabilized by weak intramolecular C7—H7···O2 hydrogen bond which generates an S(5) ring motif (Bernstein *et al.,* 1995). The crystal packing is stabilzed by intermolecular C—H···O hydrogen bonds. Atom C2 in the molecule at (*x, y, z*) donates one proton to atom O1 at (*-1+x, y, z*), forming a C(8) chain along the *a* axis (Fig. 2). The crystal packing is further stabilized by C—Cl···π interactions involving chlorine Cl1 and benzene ring (C1—C6), with a Cl···centroid(Cg^ii^) distance of 3.829 (1) Å  and a C1—Cl1···Cg^ii^ angle of 165.3 (1)° (symmetry code as in Fig. 2).

### Experimental

To a stirred solution of methyl 2-((2-chlorophenyl)(hydroxy)methyl)acrylate (4.42 mmol, 1g) in dichloro methane (DCM) was added a 48% hydrobromic acid (8.84 mmol, 0.71 g) solution and then a concentrated sulphuric acid solution (catalytic amount) at 273 K. After stirring overnight at room temperature, the mixture was diluted with DCM and water. The aqueous phase was extracted twice with DCM. The combined organic phase was washed twice with water an then dried with sodium sulphate. Removal of the solvent led to the crude product which was purified through a pad of silica gel (100—200 mesh) using ethylacetate and hexanes (1:9) as solvents. The pure title compound was obtained as a colorless solid (1.14 g, 90%). Single crystals suitable for X-ray diffraction were obtained by slow evaporation of an ethylacetate solution at room temperature.

### Refinement

All the H atoms were positioned geometrically, with C—H = 0.93 - 0.98 Å and constrained to ride on their parent atom, with *U*_iso_(H)=1.5*U*_eq_ for methyl H atoms and 1.2*U*_eq_(C) for other H atoms.

### Crystal data

**Table:**

C_11_H_10_BrClO_2_	*F*(000) = 576
*M**_r_* = 289.55	*D*_x_ = 1.661 Mg m^−^^3^
Monoclinic, *P*2_1_/*c*	Mo *K*α radiation, λ = 0.71073 Å
Hall symbol: -P 2ybc	Cell parameters from 3345 reflections
*a* = 10.0657 (7) Å	θ = 2.0–29.9°
*b* = 10.2174 (7) Å	µ = 3.76 mm^−^^1^
*c* = 11.3598 (7) Å	*T* = 293 K
β = 97.649 (2)°	Block, yellow
*V* = 1157.91 (13) Å^3^	0.24 × 0.22 × 0.16 mm
*Z* = 4	

### Data collection

**Table:**

Bruker APEXII CCD diffractometer	3336 independent reflections
Radiation source: fine-focus sealed tube	2139 reflections with *I* > 2σ(*I*)
graphite	*R*_int_ = 0.034
Detector resolution: 10.0 pixels mm^-1^	θ_max_ = 29.9°, θ_min_ = 2.0°
ω scans	*h* = −14→14
Absorption correction: multi-scan (*SADABS*; Sheldrick, 1996)	*k* = −14→14
*T*_min_ = 0.390, *T*_max_ = 0.548	*l* = −15→15
14580 measured reflections	

### Refinement

**Table:**

Refinement on *F*^2^	Primary atom site location: structure-invariant direct methods
Least-squares matrix: full	Secondary atom site location: difference Fourier map
*R*[*F*^2^ > 2σ(*F*^2^)] = 0.034	Hydrogen site location: inferred from neighbouring sites
*wR*(*F*^2^) = 0.089	H-atom parameters constrained
*S* = 0.99	*w* = 1/[σ^2^(*F*_o_^2^) + (0.0394*P*)^2^ + 0.4461*P*] where *P* = (*F*_o_^2^ + 2*F*_c_^2^)/3
3336 reflections	(Δ/σ)_max_ = 0.001
137 parameters	Δρ_max_ = 0.57 e Å^−^^3^
0 restraints	Δρ_min_ = −0.54 e Å^−^^3^

### Special details

**Table:**

Geometry. All esds (except the esd in the dihedral angle between two l.s. planes) are estimated using the full covariance matrix. The cell esds are taken into account individually in the estimation of esds in distances, angles and torsion angles; correlations between esds in cell parameters are only used when they are defined by crystal symmetry. An approximate (isotropic) treatment of cell esds is used for estimating esds involving l.s. planes.
Refinement. Refinement of F^2^ against ALL reflections. The weighted R-factor wR and goodness of fit S are based on F^2^, conventional R-factors R are based on F, with F set to zero for negative F^2^. The threshold expression of F^2^ > 2sigma(F^2^) is used only for calculating R-factors(gt) etc. and is not relevant to the choice of reflections for refinement. R-factors based on F^2^ are statistically about twice as large as those based on F, and R- factors based on ALL data will be even larger.

### Fractional atomic coordinates and isotropic or equivalent isotropic displacement parameters (Å^2^)

**Table:**

	*x*	*y*	*z*	*U*_iso_*/*U*_eq_	
Br1	0.83211 (3)	0.03430 (3)	0.63091 (2)	0.06228 (12)	
Cl1	0.43126 (7)	0.24881 (8)	1.00168 (6)	0.06382 (19)	
O2	0.91325 (15)	0.14097 (17)	1.01998 (13)	0.0471 (4)	
C1	0.4117 (2)	0.1427 (2)	0.8812 (2)	0.0437 (5)	
C6	0.5234 (2)	0.0869 (2)	0.84130 (19)	0.0391 (5)	
C7	0.6602 (2)	0.1162 (2)	0.89910 (19)	0.0381 (5)	
H7	0.6767	0.1091	0.9814	0.046*	
C8	0.7621 (2)	0.1522 (2)	0.84211 (18)	0.0358 (4)	
C10	0.8961 (2)	0.1833 (2)	0.90868 (18)	0.0387 (5)	
O1	0.98111 (17)	0.2408 (2)	0.86463 (16)	0.0634 (5)	
C9	0.7514 (2)	0.1773 (2)	0.71290 (19)	0.0439 (5)	
H9A	0.7964	0.2589	0.6994	0.053*	
H9B	0.6578	0.1863	0.6804	0.053*	
C5	0.4999 (3)	0.0026 (2)	0.7449 (2)	0.0504 (6)	
H5	0.5724	−0.0379	0.7171	0.061*	
C2	0.2834 (2)	0.1184 (3)	0.8258 (2)	0.0552 (6)	
H2	0.2102	0.1580	0.8533	0.066*	
C3	0.2648 (3)	0.0354 (3)	0.7298 (3)	0.0602 (7)	
H3	0.1786	0.0187	0.6922	0.072*	
C11	1.0420 (2)	0.1709 (3)	1.0877 (2)	0.0585 (7)	
H11A	1.1113	0.1245	1.0547	0.088*	
H11B	1.0416	0.1447	1.1688	0.088*	
H11C	1.0583	0.2633	1.0845	0.088*	
C4	0.3727 (3)	−0.0228 (3)	0.6893 (3)	0.0576 (7)	
H4	0.3599	−0.0792	0.6246	0.069*	

### Atomic displacement parameters (Å^2^)

**Table:**

	*U*^11^	*U*^22^	*U*^33^	*U*^12^	*U*^13^	*U*^23^
Br1	0.06623 (19)	0.0758 (2)	0.04610 (15)	−0.00412 (14)	0.01240 (12)	−0.01212 (13)
Cl1	0.0517 (4)	0.0854 (5)	0.0558 (4)	0.0079 (3)	0.0125 (3)	−0.0115 (3)
O2	0.0394 (8)	0.0644 (11)	0.0359 (8)	0.0020 (8)	−0.0008 (6)	−0.0001 (7)
C1	0.0385 (11)	0.0485 (14)	0.0449 (12)	0.0006 (10)	0.0091 (9)	0.0080 (10)
C6	0.0320 (10)	0.0409 (12)	0.0442 (11)	−0.0023 (9)	0.0042 (8)	0.0075 (10)
C7	0.0343 (10)	0.0416 (13)	0.0381 (10)	0.0038 (9)	0.0032 (8)	0.0036 (9)
C8	0.0309 (10)	0.0375 (12)	0.0383 (10)	0.0040 (9)	0.0023 (8)	0.0034 (9)
C10	0.0333 (10)	0.0448 (13)	0.0380 (10)	0.0053 (9)	0.0053 (8)	−0.0013 (9)
O1	0.0381 (9)	0.0980 (15)	0.0530 (10)	−0.0153 (9)	0.0023 (7)	0.0131 (10)
C9	0.0365 (11)	0.0537 (14)	0.0406 (11)	0.0007 (10)	0.0015 (9)	0.0071 (10)
C5	0.0447 (13)	0.0450 (14)	0.0617 (15)	−0.0032 (11)	0.0073 (11)	−0.0004 (11)
C2	0.0329 (11)	0.0706 (18)	0.0627 (15)	0.0017 (12)	0.0087 (10)	0.0129 (14)
C3	0.0400 (13)	0.0667 (18)	0.0700 (17)	−0.0132 (13)	−0.0064 (12)	0.0111 (14)
C11	0.0447 (13)	0.082 (2)	0.0447 (13)	0.0099 (13)	−0.0091 (10)	−0.0104 (13)
C4	0.0533 (15)	0.0524 (16)	0.0642 (16)	−0.0124 (13)	−0.0026 (12)	−0.0010 (13)

### Geometric parameters (Å, °)

**Table:**

Br1—C9	1.966 (2)	C9—H9A	0.9700
Cl1—C1	1.737 (3)	C9—H9B	0.9700
O2—C10	1.326 (3)	C5—C4	1.375 (4)
O2—C11	1.449 (3)	C5—H5	0.9300
C1—C2	1.381 (3)	C2—C3	1.375 (4)
C1—C6	1.389 (3)	C2—H2	0.9300
C6—C5	1.389 (4)	C3—C4	1.371 (4)
C6—C7	1.475 (3)	C3—H3	0.9300
C7—C8	1.336 (3)	C11—H11A	0.9600
C7—H7	0.9300	C11—H11B	0.9600
C8—C9	1.480 (3)	C11—H11C	0.9600
C8—C10	1.490 (3)	C4—H4	0.9300
C10—O1	1.201 (3)		
			
C10—O2—C11	115.55 (19)	Br1—C9—H9B	109.4
C2—C1—C6	121.7 (2)	H9A—C9—H9B	108.0
C2—C1—Cl1	118.14 (19)	C4—C5—C6	121.9 (3)
C6—C1—Cl1	120.10 (18)	C4—C5—H5	119.0
C1—C6—C5	116.9 (2)	C6—C5—H5	119.0
C1—C6—C7	121.3 (2)	C3—C2—C1	119.5 (2)
C5—C6—C7	121.8 (2)	C3—C2—H2	120.3
C8—C7—C6	124.9 (2)	C1—C2—H2	120.3
C8—C7—H7	117.6	C4—C3—C2	120.2 (2)
C6—C7—H7	117.6	C4—C3—H3	119.9
C7—C8—C9	124.71 (19)	C2—C3—H3	119.9
C7—C8—C10	120.97 (19)	O2—C11—H11A	109.5
C9—C8—C10	114.06 (18)	O2—C11—H11B	109.5
O1—C10—O2	123.2 (2)	H11A—C11—H11B	109.5
O1—C10—C8	122.7 (2)	O2—C11—H11C	109.5
O2—C10—C8	114.11 (18)	H11A—C11—H11C	109.5
C8—C9—Br1	111.16 (15)	H11B—C11—H11C	109.5
C8—C9—H9A	109.4	C3—C4—C5	119.7 (3)
Br1—C9—H9A	109.4	C3—C4—H4	120.2
C8—C9—H9B	109.4	C5—C4—H4	120.2
			
C2—C1—C6—C5	−1.7 (3)	C7—C8—C10—O2	16.2 (3)
Cl1—C1—C6—C5	179.72 (18)	C9—C8—C10—O2	−169.36 (19)
C2—C1—C6—C7	178.3 (2)	C7—C8—C9—Br1	−106.0 (2)
Cl1—C1—C6—C7	−0.2 (3)	C10—C8—C9—Br1	79.8 (2)
C1—C6—C7—C8	−130.5 (2)	C1—C6—C5—C4	1.4 (4)
C5—C6—C7—C8	49.6 (3)	C7—C6—C5—C4	−178.6 (2)
C6—C7—C8—C9	4.5 (4)	C6—C1—C2—C3	1.1 (4)
C6—C7—C8—C10	178.3 (2)	Cl1—C1—C2—C3	179.6 (2)
C11—O2—C10—O1	1.2 (3)	C1—C2—C3—C4	0.0 (4)
C11—O2—C10—C8	−179.3 (2)	C2—C3—C4—C5	−0.3 (4)
C7—C8—C10—O1	−164.3 (2)	C6—C5—C4—C3	−0.5 (4)
C9—C8—C10—O1	10.1 (3)		

### Hydrogen-bond geometry (Å, °)

**Table:**

*D*—H···*A*	*D*—H	H···*A*	*D*···*A*	*D*—H···*A*
C7—H7···O2	0.93	2.39	2.740 (3)	102
C2—H2···O1^i^	0.93	2.48	3.373 (3)	162

Symmetry codes:  (i) *x*−1, *y*, *z*.
